# Clinical experiences and success rates of acromegaly treatment: the single center results of 62 patients

**DOI:** 10.1186/1472-6823-14-97

**Published:** 2014-12-16

**Authors:** Mehtap Evran, Murat Sert, Tamer Tetiker

**Affiliations:** Department of Internal Medicine, Division of Endocrinology, Balcali Hospital, Cukurova University Medical Faculty, 01330 Adana, Turkey

**Keywords:** Acromegaly, Diagnosis, Treatment, GH, IGF-1, Pituitary hormone deficiency

## Abstract

**Background:**

This study aimed to report the clinical and outcome data from a large cohort of patients diagnosed with acromegaly and treated at our institution over a 20-year period.

**Methods:**

Sixty-two acromegaly patients (32 women and 30 men) treated and monitored at the endocrinology polyclinic between 1984 and 2013 were enrolled in this retrospective study. Clinical features and patients’ treatment outcomes were evaluated. A level of growth hormone (GH) of <2.5 ng/ml was considered as the criterion for remission, and the normal insulin-like growth factor (IGF) range was based on gender and age.

**Results:**

The mean age at the time of diagnosis was 38.8 ± 1.4 years, the time to diagnosis was 4.5 ± 0.3 years, and the follow-up duration was 7.3 ± 0.8 years. Among patients’ symptoms, growth in hands and feet and typical facial dysmorphism were the most prominent (92%). The number of patients with diabetes mellitus, hypertension and hyperprolactinemia were 22 (35%), 13 (21%) and 13 (21%), respectively. Microadenomas and macroadenomas were found in eight and 54 patients, respectively. A significant correlation was found between the initial tumor diameters and GH levels (p = 0.002). The mean GH and IGF-1 levels were 39.18 ± 6.1 ng/ml and 993.5 ± 79 ng/ml, respectively. Visual field defect was found in 16 patients (32%). Thirty-one patients were treated by transsphenoidal surgery. Four of these were cured, 10 patients developed postoperative anterior pituitary hormone deficiency, and one patient developed diabetes insipidus. Twenty patients were treated by transcranial surgery, of which two were cured, while 17 patients developed postoperative anterior pituitary hormone deficiency. In total, five of the patients who were not cured after surgery were given conventional radiotherapy, of which two were cured. Four of 15 patients, on whom Gamma Knife radiosurgery was performed, were cured. Biochemical remission was achieved in 32 of 52 patients who received octreotide treatment, and in two of five patients who received lanreotide treatment.

**Conclusions:**

The rate of surgical success in our patients was found to be low. This could be explained by an absence of experienced pituitary surgical centers or surgeons in our region, and the fact that most patients presented late at the macroadenoma stage.

## Background

Acromegaly is a rare condition with a prevalence of around 60 in 1,000,000 [1]. The cause in most patients is a pituitary adenoma that produces growth hormone (GH). Serum insulin-like growth factor 1 (IGF-1) concentration increases when its production is stimulated by GH over-secretion. The clinical features of the disease occur because of the peripheral effects of excessive GH and IGF-1, as well as tumor pressure. Diagnosis is made based on increased serum basal GH level, an IGF-1 level higher than normal limits for gender and age, and an absence of suppression in serum GH levels (>1.0 ng/ml) by the oral glucose tolerance test (OGTT). In acromegaly, progressive deformations occur in the body, especially in the face and extremities, the severity of which are associated with the time to diagnosis. The condition may also cause other morbidities such as arterial hypertension, cardiomyopathy, sleep apnea syndrome, diabetes mellitus (DM), menstrual irregularities, arthropathy, and peripheral neuropathy. Sometimes acromegaly occurs together with hyperprolactinemia [2]. The risk of malignancy, particularly colon cancer, is increased in acromegalic patients [3, 4]. The first treatment option for acromegaly is removal of the pituitary adenoma by an experienced surgeon at a specialist center for pituitary surgery. If surgery is unsuccessful, treatment using somatostatin analogs (SA), dopamine agonists, GH antagonist and/or Gamma Knife radiosurgery can be used [5]. For the disease to be considered under control, the basal GH level should be <2.5 ng/ml, GH levels should be <1.0 ng/ml by the OGTT, and the IGF-1 levels should be within normal limits based on the patient’s age and gender [6, 7].

In this study, we report the clinical and outcome data from a large cohort of acromegalic patients who were diagnosed and followed up over a 20-year period at our institution.

## Methods

### Patients

Sixty-two acromegaly patients (32 women and 30 men) diagnosed, treated and monitored at our endocrinology polyclinic between 1984 and 2013 were enrolled in this retrospective study. The files of the patients diagnosed before 2007 were reviewed, and their details were evaluated. Patients diagnosed after 2007 were included in the 62 patients and were followed prospectively in this study. In addition to the demographics of the patients, we reviewed time to diagnosis, age at the time of diagnosis, clinical examination findings, assessment of field of vision, follow-up time, treatment approach (surgical, radiotherapy, drug treatment and/or their combination) and the outcomes of these treatments. Postoperative pituitary hormone deficiency, tumor diameter, serum GH and IGF-1 levels based on age and gender, and GH response to oral glucose challenge were assessed. IGF-1 levels could not be measured before 2001 at our center; consequently, 21 of our patients did not have IGF-1 levels available at the time of diagnosis. This paper is undertaken in accordance with the ethical standards laid down in the 1964 Declaration of Helsinki and its later amendments. The local ethical committee of Cukurova University approved the study.

### Hormone assays

A chemiluminescence immunoassay was used to measure patients’ serum GH and IGF-1 levels (Immulite 2000, Siemens; ng/ml), and prolactin levels (DxI 800, Beckman Coulter; ng/ml). For imaging of the pituitary gland, pituitary CT was used in 10 patients, and pituitary MRI was used in 52 patients.

In our study, biochemical remission was defined as a basal GH <2.5 ng/ml, post-OGTT GH <1 ng/ml and IGF-1 level within normal limits based on age and gender [8]. Cases that showed permanent remission after a successful surgery or radiotherapy (conventional radiotherapy or Gamma Knife) were considered cured.

### Statistical analysis

Statistical analyses using the Shapiro–Wilk, Mann–Whitney U and Chi-squared tests were performed. For correlations between the groups, the Pearson correlation was used for the parameters that fitted normal distribution, and Spearman’s tests were used for the parameters that did not the fit normal distribution. The results were expressed as n (%) and SE ± mean and a p value of less than 0.05 was considered significant. SPSS-19 software was used for all statistical analyses.

## Results

### Characteristics of patients

Demographic and clinical characteristics of the patients are shown in Table 1. The most common symptoms, seen in 55/62 (92%) of patients, were transversal growth-thickening in hands and feet and facial dysmorphism (such as prominent forehead, separation of teeth, prognathism, and growth of cartilage). Additionally, hyperprolactinemia was found in 13/62 patients at the time of diagnosis, with four of these patients also presenting with galactorrhea. Visual field defect was found in 16/50 patients who underwent visual field examinations. The most common conditions that coexisted with acromegaly in patients were DM (35%) and hypertension (21%).

**Table 1:** **Demographic and clinical characteristics of acromegaly patients at the time of diagnosis**

	SE ± mean (Range)		n	(%)
Age (years)	46.2 ± 1.4 (18–75)	Number of microadenomas	8/62	(13%)
Age at the time of diagnosis (years)	38.2 ± 1.4 (8–65)	Number of macroadenomas	54/62	(87%)
Time to diagnosis (years)	4.5 ± 0.3 (1–14)	Hyperprolactinemia	13/62	(21%)
Follow-up duration	7.3 ± 0.8 (1–29)	Hypertension	13/62	(21%)
GH level (ng/ml); (n = 54)	39.18 (2.1–179)	Diabetes Mellitus	22/62	(35%)
^#^IGF-1 level (ng/ml); (n = 41)	993.5 ± 79 (262–3000)	Carpal Tunnel Syndrome	2/62	(3.2%)
IGF-1 level (n = 1)*	1700 ± 0	Menstrual irregularity	4/32	(12.5%)
IGF-1 levels (n = 4)**	1318 ± 788	Loss of libido	2/62	(3.2%)
IGF-1 levels (n = 17)***	770.5 ± 694	Galactorrhea	4/62	(6.5%)
IGF-1 levels (n = 19)****	1078 ± 1249	Headache	4/62	(6.5%)
		Typical facial findings and growth in hands and feet	55/62	(92%)

^#^IGF-1 levels (based on age and gender).

*Male, for 6–8 years; normal range of IGF-1: 110-565 ng/ml.

**Female and male, for 16–25years; normal range of IGF-1: 182-780 ng/ml.

***Female, for > 25 years; normal range of IGF-1: 123-463 ng/ml.

****Male, for > 25 years; normal range of IGF-1: 123-463 ng/ml.

### Laboratory and imaging findings of patients

**Figure 1:**
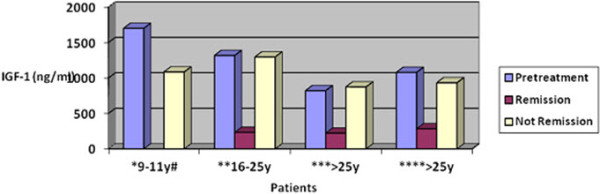
**Before and after treatment IGF-1 levels of the patients (normal range is given according to age and gender).** ^#^ y: years *Male; normal range of IGF-1: 110-565 ng/ml (n = 1), **Female and Male; normal range of IGF-1: 182-780 ng/ml (pretreatment n: 4, posttreatment n:5; remission:3) ***Female; normal range of IGF-1: 123-463 ng/ml (prereatment n:17, posttreatment n:26; remission:21) **** Male; normal range of IGF-1: 123-463 ng/ml (pretreatment n:19, posttreatment n:23; remission:13).

Patients’ GH and IGF-1 levels before treatment are shown in Table 1 and Figure 1. The patients’ pituitary CT/MRI images revealed microadenomas (<10 mm) in eight patients, and macroadenomas (>10 mm) in 54/62 (86%) patients. The tumor diameter of the patients with macroadenomas was 10–20 mm in 16 patients (25.8%), and >20 mm in 31 patients (50%). Cavernous sinuous invasion was found in 25 patients. There was a significant correlation between the initial tumor diameters and preoperative GH levels (*p* = 0.002).

### Surgical treatment methods and outcomes

Treatment methods and outcomes are summarized in Table 2. Thirty-four patients underwent surgery in our center performed by one of two surgeons, six patients underwent surgery in another center performed by the same surgeon, four patients underwent surgery in different centers by the same surgeon and the remaining 12 patients underwent surgery in four other centers performed by different surgeons. Thirty-one patients (50%) underwent transsphenoidal surgery, with four attaining postoperative cure. Three of these latter patients had microadenoma, and one had macroadenoma. Two of the 20 patients who underwent transcranial surgery attained postoperative cure. One of these had macroadenoma, and one had microadenoma. Four of the patients who did not attain cure after transsphenoidal surgery underwent transcranial surgery again. None of these patients attained postoperative cure after the second surgery. Within the patients who could not be cured following pituitary surgery, eight underwent surgery a second time, two received surgery three times and one seven times.

**Table 2:** **Rate of treatment response for different disease subtypes and treatment strategies in acromegaly patients**

	Number of patients		Number of patients responded to treatment	
	n	(%)	n	(%)
Microadenomas	8	(13%)	4	(50%)
Macroadenomas	54	(87%)	8	(15%)
Transsphenoidal surgery	31	(52%)	4	(12.9%)
Transcranial surgery	20	(32.3%)	2	(10%)
Multiple surgery	11	(17.7%)		
Radiotherapy	20	(32.2%)	6	(30%)*
Only drug treatment	6	(9.6%)	3	(50%)**
Octreotide LAR	52	(84%)	32	(61.5%)**
Lanreotide	5	(8%)	2	(40%)**
Conventional radiotherapy + ocreotide LAR	5	(8%)	2	(40%)**
Gamma Knife + ocreotide LAR	11	(17.7%)	7	(63%)**

*The number of patients who achieved cure by Gamma Knife and conventional radiotherapy.

**Biochemical remission.

### Pituitary deficiency rates

Pituitary hormones were evaluated prior to transsphenoidal surgery in only one patient, which revealed evidence of preoperative pituitary hormone deficiency in this patient. The other 61 patients were evaluated postoperatively only. In the postoperative period, one or more anterior pituitary hormone deficiencies were found in 10 patients, and permanent diabetes insipidus (DI) was found in one patient. One patient, who underwent transcranial surgery after transsphenoidal surgery, developed hypothyroidism.

In the 17 patients who underwent transcranial surgery, one or more anterior pituitary hormone deficiencies were detected during the postoperative period.

### Outcomes of radiotherapy and gamma knife radiosurgery

Conventional radiotherapy was performed on only five patients, two of whom attained cure (their durations to cure were 5 and 10 years). These five patients also received postoperative octreotide treatment. Four of the 15 patients who received Gamma Knife treatment attained cure (the duration to cure was 5 years for two patients, 4 years for one patient, and 3 years for one patient). Seven of the remaining 11 patients achieved biochemical remission with postoperative octreotide treatment. Further treatment of the patients who did not enter remission is ongoing.

### Drug treatment and outcomes

SA treatment without surgery was administered in six patients owing to the lack of eligibility or consent for surgery. Four of these patients received primary octreotide LAR, while the remaining two were given primary lanreotide. The treatment dose of octreotide was 20–30 mg/month, and normalization was achieved in serum GH and IGF-1 levels in three of the four patients. The biochemical remission durations with medication of these three patients were 6, 8 and 11 years, respectively. No remission could be achieved in two patients who received primary lanreotide treatment (90–120 mg/month).

**Figure 2:**
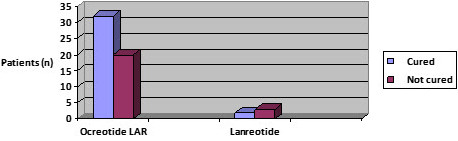
**According to pharmacotherapy remission rates of the patients.**

Forty-eight of the 62 patients who did not achieve postoperative success were given octreotide LAR treatment, and 29 of these 48 patients (60.2%) achieved remission. Likewise, a further three patients who did not achieve postoperative success were given lanreotide treatment and two of these (66.6%) achieved remission. The drug dose was increased to 120 mg for these patients (Figure 2). In total, 32 of the 52 patients (61.5%) who were given octreotide treatment, and two of the five patients (40%) who were given lanreotide treatment, achieved GH levels of <2.5 ng/ml, and IGF-1 levels within normal limits based on age (Figure 1).

### Tumor reduction

In two of the four patients who received octreotide treatment without surgery, the tumor diameter did not change (3- and 8-year follow-ups, respectively). In the other two patients, a reduction in tumor diameter was detected (from 8 to 7 mm after 6 years and from 15 to 12 mm after 12 years, respectively). In one of the two patients who received 60 mg/month of lanreotide treatment without surgery, a marked reduction in tumor diameter was observed after 6 months (from 33 to 20 mm), while in the other patient, a smaller reduction was observed after 1 year (from 11 to 9 mm).

Of the 32 patients in whom a reduction in tumor size was observed, only 20 achieved biochemical remission. Indeed, there was no significant relationship between tumor diameter and achievement of cure (p = 0.06). Interestingly, anterior pituitary hormone deficiency was found in three of eight patients with microadenoma (37.5%) and in 25 of 54 patients with macroadenoma (46.3%); this difference was statistically significant (p = 0.005; Table 3).

**Table 3:** **Frequency of pituitary hormone deficiency in the postoperative period by disease subtype**

Deficient hormones	Microadenomas (n)	Macroadenomas (n)
TSH		8
ACTH	2	
TSH + ACTH		6
FSH + LH		3
TSH + ACTH + FSH + LH		6
TSH + FSH + LH	1	2
ADH	1	
Total*	4	25

*Transsphenoidal: Anterior pituitary hormone deficiency in 10 patients, and DI in one patient.

TSH deficiency in one patient who underwent transcranial surgery after transsphenoidal surgery.

Transcranial: Anterior pituitary hormone deficiency in 17 patients.

## Discussion

Our study has made it possible to review the most important findings and challenges in managing a large cohort of acromegaly patients over a 20-year period. Although acromegaly can be seen in all ages, the average age of onset is 32 years. However, as it progresses subclinically, the diagnosis is delayed by between 4 and 10 years [1, 2]. This duration has decreased recently, for example, in a study of 100 patients, the delay in diagnosis was found to be 3.2 years [9]. More frequent MRI performed for different reasons may be one explanation for earlier incidental diagnosis of acromegaly [10]. In our patients, the age average at the time of diagnosis was found to be 38.8 ± 1.4 years, and the time to diagnosis was found to be 4.5 ± 0.3 years.

The typical features of acromegaly develop over time, and their severity is associated with a patient’s age, GH and IGF-1 levels, tumor diameter and delay in diagnosis. Indeed the delay in diagnosis can be explained, in part, by the fact that any facial changes are often attributed to aging; old photographs of the patient are therefore a useful diagnostic tool. Skeletal and soft tissue changes, organomegaly and typical facial changes are also common [11]. The most common symptoms of our patients, seen in 92%, were growth in hands and feet, and facial dysmorphism. Additionally, acromegaly patients have an increased incidence of DM, hypertension, cardiovascular diseases, breathing problems, osteoporosis and osteoarticular dysfunctions compared with the normal population. The incidence of glucose intolerance is 16–46%, and the incidence of DM is 19–56% [12, 13]. The anti-insulinergic effects of GH are considered to be responsible for the pathogenesis of acromegaly. In many cases, when acromegaly is cured, DM is also cured [14]. The most common conditions that coexisted with acromegaly in our patients were DM (35%) and hypertension (20.9%), in keeping with the literature.

The incidence of hyperprolactinemia in pituitary acromegaly patients is approximately 30–40%. The reasons for hyperprolactinemia are pressure on the pituitary stalk or simultaneous secretion of prolactin from tumor cells [15–17]. Incidence of simultaneous secretion of GH and prolactin is around 25% [18]. In our cohort, 13 patients (21%) were found to have hyperprolactinemia at the time of diagnosis, a significantly higher rate than that described in the study by Moyes et al. (13%) [19].

Around 40% of patients are diagnosed in clinics other than endocrinology for symptoms and findings of increased cranial pressure. For instance, neurological symptoms and vision problems may occur because of growth of a pituitary adenoma [11]. The incidence of visual field defect owing to increased pressure is around 19–20% in the literature [20]. The rate of visual field defect in our patients was 32%. In keeping with the published literature, imaging detected macroadenomas in 54 of our patients (87%) [7, 21].

In acromegaly, serum basal GH measurement, post-OGTT GH measurement and IGF-I levels measurement are the gold standard assays for measuring disease activity and monitoring the effectiveness of treatment. While GH measurement has become more sensitive in the recent decades, IGF-1 measurements can be misleading in situations such as malnutrition, liver disease and kidney failure [22, 23]. In particular, inconsistencies between GH and IGF-1 measurements can be seen throughout SA treatment [17]. Our patients usually showed a positive correlation between the average GH levels and IGF-1 levels at the time of diagnosis and during SA treatment.

The first treatment option in acromegaly is transsphenoidal tumor excision, particularly for intrasellar microadenomas and noninvasive macroadenomas. In situations such as patient non-consent, existence of serious cardiomyopathy and respiratory disease or absence of an experienced surgeon, then other treatment options are considered. The finding of visual field defect or neurological deficit is almost always an urgent surgical indication [24, 25]. We also apply transsphenoidal surgery as the first treatment option for many of our patients.

In a previous retrospective study of 100 patients who underwent transsphenoidal surgery with a remission criterion of GH <5 mU/l, 42% of the patients achieved postoperative remission. In this study, the remission rates were associated with tumor diameter and preoperative GH levels, and 21 patients were found to have pituitary hormone deficiency [26]. The postoperative cure rate of patients who underwent transsphenoidal surgery was 12.9%. In studies where remission criteria were considered to be either GH <2.5 ng/ml, post-OGTT GH <2 ng/ml or IGF-1 within normal limits based on age and gender, the remission rates after transsphenoidal surgery were found to be 38, 57 and 37%, respectively, and the anterior pituitary hormone deficiency rates were found to be 35, 8 and 10%, respectively [7, 27, 28]. Currently, the reported success of intrasellar surgery varies between 75 and 95% for intrasellar microadenomas, and between 45 and 68% for noninvasive macroadenomas, if undertaken by experienced surgeons who perform at least 50 case surgeries per year [24, 29]. When our patients were evaluated, four of 31 patients who were operated transsphenoidally attained postoperative cure. Three of these patients had microadenoma, and one had macroadenoma. While anterior pituitary hormone deficiency was found only in one patient before surgery, it was found in 37% of the patients after surgery. The rate of post-operative pituitary hormone deficiency was found to be lower in our cohort compared with the study by Sheaves et al., but higher than that observed by Swearingan et al. [26, 27].

Although the first approach in treatment is transsphenoidal surgery, a transcranial approach is required in some situations such as suprasellar tumor expansion. In another study with remission criteria similar to ours with a follow-up period of 19 years, 26 of 668 acromegaly patients underwent transcranial surgery. The postoperative remission rate of these was 7.7%, the anterior pituitary hormone deficiency rate was 5%, and the rate of permanent DI was 11.5%. In the latter series, 140 patients were re-operated, and the remission rate increased to 27.1% [30]. In our study, two of the 20 patients who were operated on transcranially attained postoperative cure, one of whom had macroadenoma and the other microadenoma. In the patients who did not achieve cure after transsphenoidal surgery and then underwent transcranial surgery, none attained cure, with only one developing secondary hypothyroidism. Although our data on pituitary hormone deficiency before transcranial surgery was insufficient, the rate of patients with anterior pituitary hormone deficiency after transcranial surgery was 85%, a markedly higher rate compared with similar published cohorts. Additionally, the rate of anterior pituitary hormone deficiency in our patients who underwent transcranial surgery was significantly higher than in the patients who underwent transsphenoidal surgery (p < 0.001).

The success of treatment in acromegaly is negatively correlated with tumor diameter and basal GH levels and positively correlated with the experience of the surgeon. Additionally, if the tumor is invasive, this decreases the likelihood of successful surgery [24, 31]. Indeed, when we assessed our results based on tumor size, 50% of those with microadenoma and 15% of those with macroadenoma were cured. Unfortunately, owing to small sample numbers, assessment of significance was not feasible. Precise tumor diameter was significantly associated with GH level (p = 0.002), but did not correlate with cure rate (p = 0.06). Additionally, only 20 of 32 patients whose tumor diameters reduced developed biochemical remission. Twenty-five of our patients also had cavernous sinuous invasion.

Another important factor that affects success rate is the number of surgeons who can perform this surgery, and the number of pituitary surgeries performed by the surgeons. Additionally, a greater number of years of surgical experience also increases the surgical success rate [25]. Our cure rate after transsphenoidal and transcranial surgery was very low compared with the literature. More than half of our patients (n = 34) were operated on in our center by one of two surgeons while the others were operated on in several external centers. The lack of experience of our surgeons in pituitary surgery, the absence of a single specialist operating center and the existence of macroadenoma or cavernous sinuous invasion at the time of diagnosis are therefore likely contributors to our low surgical success rate.

Radiotherapy is preferred in situations where surgery is contraindicated or unsuccessful, or when medical treatment is insufficient in controlling GH secretion [32]. An average of 60% of patients who receive conventional radiotherapy attain GH and IGF-1 normalization, but the maximum response is seen after 10–15 years [24, 33]. While conventional radiotherapy is preferred in large recidive tumors or in tumors close to the optical nerve, Gamma Knife radiosurgery is preferred in smaller tumors. The 5-year remission rates following Gamma Knife is between 29 and 60% [34]. The incidence of pituitary deficiency for both methods is similar [28, 31]. Some of our patients who did not achieve cure after surgery received radiotherapy. Thirty percent of the patients who received conventional radiotherapy or Gamma Knife attained cure, and a further 50% entered biochemical remission. The time to cure was 5–10 years for the two patients who received conventional radiotherapy, and 3–5 years in the four patients who received Gamma Knife. Thus, our rate of cure with surgical treatment and radiotherapy was 19.3% (12 patients), which is in keeping with the literature.

It has been shown *in vitro* that natural somatostatin inhibits GH secretion in many GH-secreting tumors. For this reason, SAs have been developed for treating acromegaly. Somatostatin analogs work by activating somatostatin receptors. There are, however, major gastrointestinal side effects associated with these drugs. Long-lasting forms of somatostatin analogs are preferred, and octreotide LAR and lanreotide autogel are in current clinical use in Turkey, but not pasireotide. Pegvisomant is a GH receptor antagonist, which can be used alone or in combination with SAs [17]. Somatostatin analogs can be used for situations when there is little possibility of surgical cure such as large extrasellar tumors without pressure effect, in patients who cannot be controlled biochemically with surgery, and to provide biochemical control while waiting for the effect of radiotherapy. Although there are data that show that preoperative SA use has benefits on GH and IGF-1 normalization and on postoperative hospitalization, some studies have concluded that it does not [24, 35]. Some studies have shown that with use of somatostatin analogs, biochemical remission is attained, and tumors become smaller in size [36, 37]. Several long-term retrospective studies have reviewed the effects of SA given postoperatively and/or primarily, and have reported a wide variation of biochemical remission rates of between 34 and 95% [38, 39]. Investigations comparing efficacy of lanreotide and octreotide treatments have reported a similar rate of cure of symptoms and biochemical cure for both agents [40–42].

It should be kept in mind that our study is not a study for evaluating response to primary SA treatment. In our study, we used serum GH <2.5 ng/ml and IGF-1 normalization as biochemical remission criterion, and the serum GH and IGF-1 levels of our patients who received drug treatment decreased significantly compared with the baseline (p < 0.001). When we review all our patients with regards to biochemical remission, we see that 32/52 patients (61.5%) who received octreotide treatment and 2/5 patients (40%) who received lanreotide treatment are in biochemical remission. However, it should be noted that 10 of the patients who achieved biochemical remission received Gamma Knife, and five received conventional radiotherapy; therefore, the cause of remission may be multifactorial in these patients.

In a recent study by Coloa et al., which followed up 45 acromegalic patients, no improvement in glucose intolerance or DM prevalence was seen [37]. However, we observed improvement in DM in four of the 22 patients in our cohort who were initially diagnosed with DM. Two of these patients, whose blood glucose levels improved were in remission and one of them was cured.

A notable restriction of our study was that the file archiving system was more irregular and insufficient in previous years, and therefore we did not have access to a full range of data for all patients. For example, the initial IGF-1 level was the most important predictor of adequate response to treatment [43]; however, this measurement was not available for some patients. Furthermore, we did not have access to data concerning presence or absence of pituitary hormone deficiency before treatment. Additionally, the majority of our patients were operated on in different centers by different surgeons and some patients did not attend our hospital for assessment with optimum regularity. Indeed, infrequent monitoring may be an additional contributing factor to the low rate of cure in our cohort compared with the literature.

In conclusion, we believe the rate of successful treatment of acromegaly will increase with earlier diagnosis, greater surgical experience and regular and appropriate follow-up after surgery. Future prospective studies of large cohorts will help to provide further information on appropriate treatment strategies in this disease.
